# Applying an AI Decision Support Device During Cervical Cancer Screening in Bangladesh and Uganda: Implementation Study

**DOI:** 10.2196/81493

**Published:** 2026-09-03

**Authors:** Jaap Koot, Johnblack Kabukye, Guruvare Shyamala, Naheed Nazrul,, Aminur Rahman Shaheen, Carolyn Nakisige, Shamim Namagembe, Keerthana Prasad, Jogchum Beltman

**Affiliations:** 1 Global Health Unit, Department of Health Science, University Medical Center Groningen, Hanzeplein 1, Groningen, 9713 GZ, The Netherlands, 31 622487689; 2 Uganda Cancer Institute, Kampala, Uganda; 3 Department of Gynecology and Obstetrics, Manipal Academy of Higher Education, Kasturba Medical College, Manipal, Manipal, India; 4 Friendship, Dhaka, Bangladesh; 5 Health Systems and Population Studies Division, International Centre for Diarrhoeal Disease Research, Dhaka, Bangladesh; 6 Public Health Department, The University of Comilla, Dhaka, Bangladesh; 7 Uganda Rural Development and Training Program, Kigadi, Uganda; 8 Manipal School of Information Sciences, Manipal Academy of Higher Education, Manipal, India; 9 Department of Obstetrics and Gynaecology, Leiden University Medical Center, Leiden, The Netherlands

**Keywords:** cervical cancer screening, visual inspection, artificial intelligence, device, Consolidated Framework for Implementation Research, CFIR, implementation, barriers and facilitators

## Abstract

**Background:**

Screening is important for early detection of cervical cancer in low- and middle-income countries. Visual inspection with acetic acid (VIA) is usually the method of choice in these settings. However, interpretation of VIA results is subject to interobserver and intraobserver variability. AI decision support systems (AI-DSSs) could contribute to better decisions by health workers.

**Objective:**

The aim of this study was to analyze the barriers and facilitators of introducing an AI-DSS device under field conditions in the context of VIA screening in rural Bangladesh and Uganda, with the goal of improving the operational systems of applying an AI-DSS device.

**Methods:**

We operationalized the Consolidated Framework for Implementation Research for this specific study and defined the constructs for analysis. The study was performed in rural Uganda and Bangladesh. We extracted relevant information from routine data, patient surveys, and facility surveys. We also interviewed health workers who used the AI-DSS devices. A panel of experts performed an analysis of the quality of pictures and the performance of health workers. We monitored implementation through quarterly meetings and documented the process. This trial was registered under ClinicalTrials.gov identifier NCT05234112.

**Results:**

The applied AI-DSS passed tests under laboratory conditions but performed less well under field conditions. The hardware design using an adapted mobile phone was adequate, and the user interface was user-friendly and intuitive. However, operating the device while performing VIA in clinics was challenging. Since the device was not registered as a medical device, it was only used for research purposes. In both countries, there was no official government policy concerning the use of AI in health care. All facilities had gynecological examination rooms, but some facilities did not have permanent electricity or internet connection. The normal procedure for VIA was followed, and AI-DSS did not interfere with routine screening procedures. The AI-DSS was well accepted by women when privacy was guaranteed, but there seemed to be more trust in the judgment of health workers. The pictures helped supervisors to give a second opinion from a distance and were used for training purposes. The technical support team was able to help remotely and improved the performance. Training health workers in taking good pictures was very important. A permanent monitoring process during implementation was established, which led to early detection and correction of shortfalls.

**Conclusions:**

According to this study, the device has the potential for improving the quality of the assessment by health workers who perform VIA. However, competent staff trained in performing VIA will be indispensable for capturing high-quality pictures. The algorithm requires further fine-tuning. This study showed that the Consolidated Framework for Implementation Research is a proper tool for categorizing barriers and facilitators when introducing an AI-DSS device in practice.

## Introduction

### Cervical Cancer Screening

With an estimated 604,000 new cases and 342,000 deaths worldwide in 2020, cervical cancer is the fourth most frequently diagnosed cancer and the fourth leading cause of death from cancer among women. More than 85% of these women with cervical cancer live in low- and middle-income countries (LMICs) [1,2].

Nearly all cervical cancers are caused by an infection with human papillomavirus (HPV), which can be significantly reduced by HPV vaccination (primary prevention). However, since less than 30% of LMICs have a national HPV vaccination program [3], secondary prevention (ie, screening and treatment of precancerous lesions) remains the most important instrument in reducing the incidence of cervical cancer. In LMICs, visual inspection with acetic acid (VIA) is often the preferred method of screening [4].

In 2021, the World Health Organization (WHO) updated its guidelines for cervical cancer prevention and recommended HPV testing as the primary screening test because of its objectivity, high sensitivity, cost-effectiveness, and the possibility for self-sampling. VIA is recommended as an option for triage following a positive high-risk HPV (hrHPV) test to identify women’s eligibility for treatment of these precancerous lesions [5]. However, performance and diagnostic accuracy of screening programs based solely on VIA vary widely and often depend on the experience of the providers, with high intraobserver and interobserver variation [6] and low sensitivity and specificity (ranging from 62.5% to 80% and from 80% to 98.8%, respectively) for the detection of cervical intraepithelial neoplasia grade II or more advanced cervical lesions against the reference standard of histology or colposcopy followed by biopsy [7-9]. VIA is therefore associated with the potential risk of overtreatment and sometimes misses high-risk cases for cervical cancer [10]. Additional factors besides the experience and skill level of the provider that may contribute to inconsistent results of VIA are the light source and the quality of the acetic acid solution. The WHO guidelines offer countries the choice to perform presumptive treatment of HPV-positive women with cervical ablation to overcome issues of missed diagnosis in VIA.

### AI Decision Support System

To overcome the limitations of intraobserver and interobserver variation in detecting precancerous lesions, AI has increasingly been applied in medical imaging, including cervical cancer screening [11]. Several studies in both high-income countries and LMICs have demonstrated that the use of AI in VIA testing, cytology, or colposcopy improved detection rates of precancerous lesions with good accuracy [12]. The use of AI therefore could support the decision-making of providers who are performing VIA, with the potential of reducing intraobserver and interobserver variability [13]. However, most studies were carried out with existing pictures or in academic hospitals. No study was implemented in remote rural areas.

In this article, we share our analysis of the introduction of a new AI decision support system (AI-DSS) device under field conditions in women undergoing cervical cancer screening in 2 LMICs with the use of VIA after being tested HPV positive, which we carried out on the basis of secondary data after completion of the project. The goal of this study was to identify relevant factors of the implementation process and to understand barriers and facilitators in using an AI-DSS device in low-resource settings.

## Methods

### Analytic Framework

**Figure 1.**
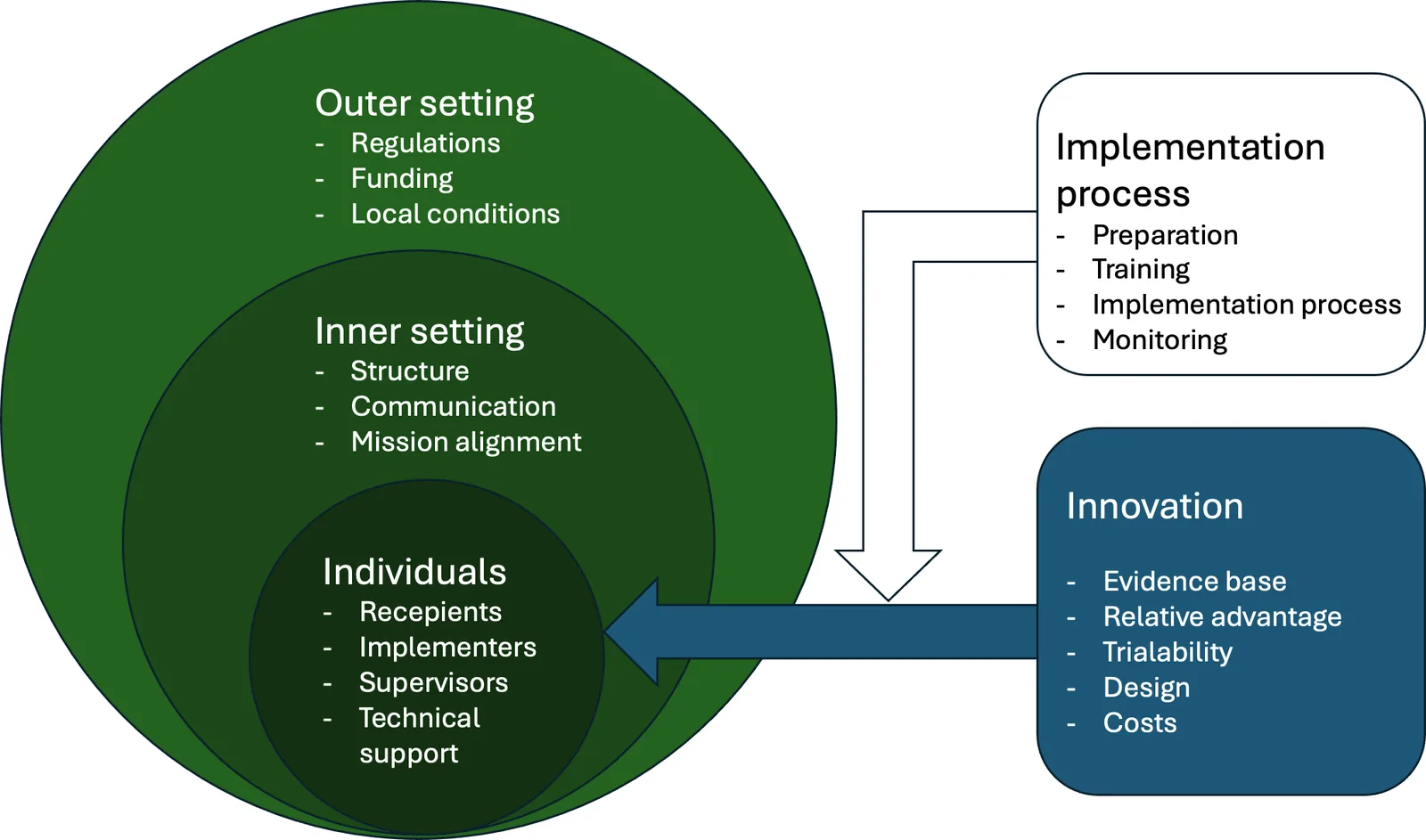
Consolidated Framework for Implementation Research framework domains of innovation, outer and inner setting, individuals, and implementation process; descriptions and constructs in each of the domains, shown in their interactions among domains, which were analyzed in this study. Adapted from Damschroder et al [14].

The Consolidated Framework for Implementation Research (CFIR) provides a structured, comprehensive method for analyzing various enablers and challenges of implementing and scaling up health interventions [14]. Factors are evaluated across 5 standard domains in CFIR (innovation, process, outer setting, inner setting, and individuals), each with multiple constructs and topics (Figure 1, adapted from Damschroder et al [14] and Toolbox [15]).

In our analysis, the CFIR domain “innovation” refers to the AI-DSS device, and the constructs are evidence base, relative advantage, trialability, design, and costs. The CFIR domain “implementation process” describes the activities that have taken place at the various study sites during the PRESCRIP-TEC (Prevention and Screening Innovation Project Towards Elimination of Cervical Cancer) project, with the constructs of preparation, training, implementation process, and monitoring. The CFIR domain “outer setting” is defined as the health system and health facility in which the cervical cancer screening takes place. The constructs are regulations, funding, and local conditions. The CFIR domain “inner setting” is the cervical cancer screening activity within the facility, with the constructs structure, communication, and mission alignment. The CFIR domain “individuals” consists of persons involved in various positions related to the use of AI-DSS, such as recipients, implementers, supervisors, and the technical support team.

### Study Setting

For this study, an AI-DSS for interpreting VIA results (called SAKHI Manipal), developed by Manipal Academy of Higher Education (MAHE) in India, was introduced in Uganda and Bangladesh as part of a European- and Indian-funded research project on cervical cancer screening, the PRESCRIP-TEC, implemented from 2021 onward [16]. In this project, academic institutions and nongovernmental organizations (NGOs) from Uganda and Bangladesh collaborated with European and Indian universities and NGOs.

In Bangladesh, the research was conducted by the research institute icddr,b and the local NGO Friendship. HPV self-collected tests were offered at home and analyzed in a Friendship laboratory. For HPV-positive women, VIA with possible treatment was provided in the floating hospitals of Friendship in Kurigram, Gaibandha, Bogura, and Sirajganj districts in the North and Sathkhira district in the South, which are remote riverine nomadic islands (chars) and coastal areas.

In Uganda, the research was implemented by the Uganda Cancer Institute and the local NGO Uganda Rural Development and Training in 1 rural district. HPV tests were collected at home and analyzed in a local laboratory. VIA triage of HPV-positive women was performed in governmental health centers. Study sites differed among each other in terms of hrHPV prevalence, risk factors for HPV infection, and organization of the cervical cancer screening program.

In each country, 8000 women were targeted for cervical cancer screening, but only hrHPV-positive women were invited for VIA triage. In total, 1183 hrHPV-positive women (136 women in Bangladesh and 1047 in Uganda) underwent VIA triage supported by AI-DSS.

### Participants

Women eligible for cervical cancer screening participated in different surveys, as explained below. In total, 5 midwives in Bangladesh and 10 midwives in Uganda performed VIA triage in combination with AI-DSS. Before starting the work, the midwives were given a 3-day practical and theoretical training session on the study protocol, including a refresher on VIA, HPV testing, and how to use the AI-DSS. During supervision visits, further training was provided twice per year. The work was regularly reviewed with the midwives during the project.

In each country, a physician with expertise in VIA triage provided regular supervision and could be consulted remotely or directly on difficult cases, as deemed by the screening nurse, or when there was discordance between the nurse and the AI-DSS. The number of consultations was not recorded, as the physician was often present in the examination room. Four gynecologists from a Dutch NGO (Female Cancer Foundation) provided field supervision once per year.

For the assessment of the quality of images captured by midwives, 6 gynecologists from Bangladesh, Uganda, India, and the Netherlands participated in a reference panel.

### Operationalization of the CFIR Framework and Data Collection

The CFIR framework (Figure 1) lists the constructs in each domain. We extracted data for each domain of this CFIR analysis from studies in the PRESCRIP-TEC project in Bangladesh and Uganda. Multimedia Appendix 1 provides an overview of the sources of information per domain and construct. Relevant information was extracted from routine data of participating patients (clinical data and data generated by the AI-DSS). Additionally, data were recorded from 3 types of surveys, which provided information about perceptions of patients and AI-DSS users (the adapted African Women’s Awareness about Cancer survey [17], the Health Service Trust Framework survey [18], and the preintervention and postintervention surveys developed in the project). Questionnaires were pretested and validated, translated, and back-translated. The WHO standardized Service Availability and Readiness Assessment survey for cervical cancer services [19] provided information about the health facilities. Country-specific model-based cost-effectiveness and budget impact analyses were conducted for the 2 countries, comparing the PRESCRIP-TEC strategy with the existing screening strategy in each setting. Data from initial project implementation informed the relevant model parameters. Costs were expressed in national currency units of each country, as well as 2022 US dollars. The results from the cost-effectiveness analyses were used to estimate the 5-year budget impact for defined target populations in each country [20].

A semistructured questionnaire was developed to evaluate experiences of health providers who worked with the AI-DSS device. Multimedia Appendix 2 provides more details about the quantitative data collection methods and results.

Local enumerators conducted in-depth interviews with 10 health workers (5 in Uganda and 5 in Bangladesh).

A logbook of evaluation meetings conducted with country managers supervising the health providers using the AI-DSS was updated quarterly. From the meeting minutes, we extracted relevant information, especially concerning the process. The project coordinator, the MAHE design team, and country implementation teams performed 2 online evaluation meetings in the beginning and midway through the implementation process with users of the AI-DSS.

Before the start of AI-DSS implementation, the skills of the selected health workers who would perform VIA were assessed [21].

An analysis of the quality of images and the performance of health providers applying VIA was performed using a panel of 6 expert gynecologists who assessed 285 of a total of 1183 preimages and postimages collected during the VIA procedures and provided a consensus reference assessment of VIA-positive (abnormalities of the cervix) or VIA-negative (no lesions identified). In total, 38 pictures out of 285 were rejected because of quality issues. Every third picture taken per country was assessed until September 2023. Pictures taken after that time were not included. First, the panel members analyzed pictures individually, and when no majority opinion was found, a consensus meeting was conducted. The MAHE design team also assessed the pictures to evaluate whether they met the quality criteria as described in the user manual of the AI-DSS (visibility of cervix, lighting, and reflection of speculum).

### Data Analysis

The authors aggregated incoming data and information during the project. Research assistants transcribed or summarized the interviews, which were later coded by the authors according to constructs in the CFIR framework. The research team performed regular analyses of facilitators and barriers using a learning-by-doing approach during the implementation research and made recommendations for improvement of the process.

In a consortium meeting in November 2023, a qualitative analysis of facilitators and barriers was performed for the introduction of the AI-DSS in practice.

### Ethical Considerations

This study was conducted in accordance with the ethical principles outlined in the Declaration of Helsinki. Patients and individual health providers provided written or electronic consent for the use of the information they provided in the research. Participants did not receive any financial compensation for participation in the study.

There was a strict separation between the medical data and research data. Patient data collected were not accessible to researchers. Data managers in the health services removed all personal patient data. Researchers received anonymized data with a random number per patient, which consisted of preimages and postimages, a data file with a random number, date of picture, code for device, judgment by health care worker, and judgment by AI-DSS.

There was role-based access control for all AI-DSS and mobile health (mHealth) app devices and computers used for data management, with a dedicated IT officer overseeing this access control, opening accounts for users, etc. The AI-DSS, an Android phone, was modified to ensure no other apps could be installed besides the AI-DSS algorithm by disabling the SIM card, Wi-Fi access, and other connections. The devices were kept in the clinic at all times.

The mHealth app used and servers used TLS/SSL encryption when transmitting data.

Participants were assigned participant ID numbers, which were used in place of identifying personal information in the research data. These IDs were kept in a separate master log where linkage to identifying information (names, addresses, phone numbers, etc) could be obtained for follow-up and clinical management of the patients as needed. The research team did not have access to the master log, except CN and SN, who were also involved in the clinical care of the participants. Furthermore, the cervical images were assigned a random number generated by the AI-DSS.

All members of the study team underwent research ethics training on human subject protection and good clinical practice.

This study received ethical clearance from relevant institutions in the participating countries: Bangladesh, icddr,b approval 21029; Uganda, National Council for Science and Technology HS2222ES. MAHE received ethical clearance for the AI-DSS study from Kasturba Medical College IEC 924/2020.

This trial was registered under ClinicalTrials.gov Identifier NCT05234112.

## Results

This section describes the findings of the CFIR analysis per domain and per construct as described in Figure 1.

### The Innovation AI-DSS

#### Evidence Base

The AI-DSS was developed by MAHE and contains several image processing algorithms built using MATLAB R2017a and later implemented in an Android device (see below, *Hardware*). A binary support vector machine VIA image classifier algorithm was trained on 102 cervical images (42 positive and 60 negative) annotated by a single cervical cancer screening expert. It was validated using k-fold and leave-one-out cross-validation. The developed algorithm achieved an accuracy of 100 (98%), with a sensitivity of 101 (99%) and specificity of 99 (97%). Based on prior scientific publications, it was considered robust enough to start the evaluation under field conditions [12]. The algorithm was set to produce a red light (further examination required) or a green light (no cervical cancer or precancerous lesions detected). Further examination included anomalies of the cervix that could not be cervical cancer, such as chronic infections or polyps.

#### Relative Advantage

Given the evidence on interpersonal and intrapersonal variation, it was expected that the AI-DSS would give more consistent results than VIA performed by health care workers.

#### Design

##### Hardware

The AI-DSS consisted of a Motorola E7 Power mobile smartphone, with an inbuilt light source next to the 13-megapixel camera, 4 GB of RAM, and 64 GB of storage capacity. The battery of the mobile device allowed for use of the device during periods of absence of electricity. With the algorithm installed on the device, the AI-DSS could be used offline. The SIM card was disabled, as was the use of Wi-Fi, to ensure patient privacy.

##### User Interface

**Figure 2.**
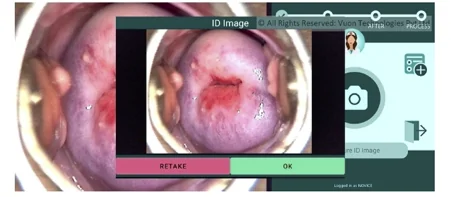
Screenshot of the SAKHI Manipal device, which was used as the AI decision support system device in this study, showing the stage in which the health worker approves the cervical image during visual inspection with acetic acid. The Manipal Academy of Higher Education obtained written consent from the person from whom this image was acquired to publish it in the instruction manual.

MAHE published an online manual to provide detailed information on how to operate the device (specially prepared mobile phone) [22]. The user interface (UI) was in English and worked mainly with pictograms. It was designed to guide the health care worker through the process of identification of the patient, prepicture, postpicture, assessment, and storage, and had a process status bar that marked the steps in the process. The UI offered options such as zooming and operating the light source. Before storing the prepictures and postpictures, users were asked to check whether the cervix was centered and the squamous-columnar junction (SCJ) was fully visible. If not up to standard, it offered the option of retaking images (Figure 2). After capturing a picture, the UI provided options for storage. The application automatically produced a folder with 3 images, that is, an image of the woman’s ID (or other patient-specific information, eg, a patient clinic number), and prepictures and postpictures when coloring with acetic acid. The prepictures and postpictures had a unique code imprinted, which could not be removed. The application produced a text file with the relevant information on the device used, the date of the examination, a random number for the patient, and the health care worker’s assessment and AI algorithm’s result. This downloadable folder was used for evaluation and research purposes.

In the quarterly evaluation interviews and meetings on user-friendliness of the device, the health providers were positive about the performance of the device (eg, the light source, the intuitive UI in guiding them through the steps during the screening, and the ability to use the device comfortably after a 3-day initial training).

On the other hand, providers reported that keeping the device steady while performing VIA triage and operating the UI simultaneously, especially in zooming mode, was a challenge. Keeping the speculum focused on the cervix while simultaneously zooming and capturing high-quality images was not considered an easy task. MAHE pretested a holder for the device mounted onto the speculum but found that the total weight became too heavy. As an intermediate solution, the device was operated by an assistant while the health care worker was performing the VIA.

Another issue encountered by users in Bangladesh and Uganda was that the maximum interval of time (30 min) allowed between the precoloring picture and the postcoloring picture initially created problems in some cases (3 incidents were reported). Pictures and incomplete folders were deleted when more time was needed. In 3 cases, this resulted in failure to complete the procedure. Health care workers were better instructed to stay within the time limits.

Transferring pictures could only be done by connecting the device to a computer using a USB cable. This required the availability of a computer at the location where the VIA was performed. Therefore, if not available in health facilities, computers were provided by the project. IT experts from Friendship in Bangladesh and Rural Development and Training in Uganda helped when needed.

In interviews, health care workers expressed concerns about keeping the device clean, as only mild detergents could be used on telephones. In practice, no problems were encountered with the cleanliness of the devices, as there was no contact between the device and patients’ bodies.

### Costs

The costs per device were around US $600, which were the costs of the adapted mobile phone purchased by the PRESCRIP-TEC project, not including the costs of developing and maintaining the algorithm software and UI. No additional costs for the devices were incurred during the project.

### Outer Setting (Health System and Health Facilities)

#### Regulations

The European Union requires a Conformité Européenne mark before the introduction of a medical device [23]. In Bangladesh and Uganda, government agencies such as the National Drug Agency must provide permission for the use of medical devices. Since the AI-DSS was not registered as a medical device in either country during this stage of field testing, the health care worker’s assessment led the decision-making, not the AI-DSS. Health care workers could not see the AI-DSS verdict before entering their own findings in the device. The AI-DSS assessment was used solely for comparison purposes. Research was the primary focus of the AI-DSS implementation at this stage. Permission was granted by national and local health authorities to conduct the research; however, in neither country was there an existing policy regarding the use of AI-DSS in cervical cancer screening.

#### Local Conditions

The VIA and AI-DSS were performed in existing health facilities. The device was designed for use in resource-constrained situations, where electrical supply and internet access are not always stable. It worked well under these conditions. However, for connecting to the internet for a second opinion or uploading files for scientific reasons, a computer with electricity and internet connectivity was required. Therefore, traveling from clinics to institutions or offices with electricity and internet was sometimes needed. Eventually, this delayed the second-opinion process in 3 or 4 cases.

#### Funding

The funding for this research, for salaries, equipment, and materials, was part of the grant from the European Union and the Indian Government, and neither health services nor patients were asked to contribute financially. In the project, no detailed analysis of the costs associated with using the device was conducted, as it was fully integrated into performing VIA.

### Inner Setting (The Screening Activity)

#### Structure

VIA screening is a standard procedure in cervical cancer screening in Bangladesh and Uganda and is part of national protocols and standard operating procedures. However, due to financial and organizational constraints in these countries, cervical cancer screening is generally opportunistic or project-based. Therefore, not all health facilities were fully prepared for performing VIA.

For using the AI-DSS, no additional materials or supplies were required, except for the standard requirements for VIA (examination bed, speculum, acetic acid coloring, and consumables such as swabs). In the interviews, some respondents complained about the availability of acetic acid, swabs, or poor infrastructure, which delayed the study.

#### Communication

Prior to screening, women were informed about the use of the AI-DSS, with a particular emphasis on the use of a camera. All participants provided written consent prior to the initiation of the VIA.

#### Mission Alignment

The AI-DSS integrated smoothly into the screening procedure, requiring only a few additional steps in the workflow. As mentioned before, in most settings, an assistant operated the AI-DSS device, while the nurse or midwife performed the VIA. The process of taking pictures only added a few extra minutes to the normal VIA procedure.

### Individuals

#### Recipients

According to health care workers interviewed, women were often apprehensive about their privacy during the screening process because a mobile phone was involved. Explaining the purpose and showing the cervical pictures was reassuring and often appreciated by women. In the Health Service Trust Framework survey, 98.9% (1246/1259) of the women participating in the survey in both Uganda and Bangladesh indicated that they trusted the treatment provided during cervical cancer screening using the device. However, only around 49.0% (127/259) of the women in Uganda reported trusting the assessment by an AI-DSS. They relied more on the health care worker’s opinion.

#### Implementing Health Care Workers

In each country and institution, health providers (nurses, midwives, and medical doctors) were trained in VIA and the use of the AI-DSS device. Those already conversant with VIA received an additional 3 days of training in applying the AI-DSS device. In Bangladesh and Uganda, we assessed the baseline quality of VIA assessment of trained health care workers prior to this study by measuring their diagnostic performance against already existing VIA images presented on a computer screen. Results showed that the diagnostic performance of health care workers was adequate in both countries. Sensitivity (compared with the expert panel assessment) was 80% (28/35) and specificity was 81.2% (39/48) [21]. During the project, the performance of the health care workers was assessed again under field conditions against the panel opinion of gynecologists who are experts in VIA. The sensitivity was 62.5% (20/32) and the specificity was 79.1% (171/216), which was slightly less than the baseline measurement.

Multimedia Appendix 3 provides confusion matrices, accuracy, sensitivity, specificity, and Cohen κ for both the AI-DSS and the health care workers. Both health care workers and AI-DSS demonstrated a high negative predictive value of over 90%, indicating that if no anomaly was found, the likelihood was high that the gynecologists’ panel would agree. The positive predictive value was lower (31% for health care workers and 20% for the AI-DSS), which means that too often cervical cancer was suspected when, in the view of the panel, it was not present. For the AI-DSS, the algorithm was calibrated to be on the safe side. A positive assessment was inviting further diagnosis, not a certain cervical cancer diagnosis. Also, cervicitis or polyps produced a red-light warning. Mucus or blood on the cervix did too.

In interviews, health care workers mentioned that using the AI-DSS device required training. Assessing whether the picture was up to standard for AI assessment (full cervix with SCJ visible) was not always easy for health care workers during the VIA process. They did not want to make the patient wait. Additional training, instructional videos, and improvements to the troubleshooting section in the manual helped increase the self-confidence of health care workers in operating the device.

Health care workers were reassured when, after their own assessment, the AI-DSS provided a similar outcome; however, they were concerned when the AI-DSS disagreed with their own assessment. This was the case in around 21.4% (54/252) of the total assessments. Health care workers indicated that the presence of blood on the cervix often led to a positive assessment by the AI-DSS, whereas they were convinced it was negative. When the AI device provided a positive result while the health care worker assessed it as negative for precancerous lesions, health care workers often discussed the case with their supervisors.

#### Supervisors

The supervision of health care workers by gynecologists varied per country. In Bangladesh, the VIA was performed in a facility that always had a medical doctor with experience nearby who could be consulted in case of problems with the device. In Uganda, the gynecologist was often not physically present. For remote consultation, the gynecologist could be reached by telephone. Pictures could be sent to the expert, who could respond immediately. Because the Wi-Fi function was disabled in the device, pictures had to be transferred via a USB cable to the computer and sent to the supervisor via an encrypted message in mHealth. Consultation by phone took place in such cases. It required that the women involved had to wait for the final result of the second opinion.

In Bangladesh and Uganda, a team of gynecologists and VIA experts from the Netherlands visited each research site twice during the project to provide technical support, helping to resolve operational problems.

#### Technical Support Team

The MAHE team consisted of 4 experts (in information sciences and in gynecology). They developed the training manual and provided constant technical support via a hotline to the local teams, as well as organized distance-learning training sessions. Via remote control, the team could update the software in devices in the 2 countries when devices were connected to a computer. Unfortunately, it was not possible for the support team to visit Bangladesh and Uganda, partly because of COVID-related travel restrictions. Upcoming issues were discussed online with the local teams and the project coordinator, and solutions were found.

### Implementation Process

#### Planning/Doing/Tailoring Strategies

The MAHE AI-DSS development team was responsible for the AI-DSS software and hardware. In particular, the development of the hardware and UI software took time due to delayed funding, while in Bangladesh and Uganda, hrHPV testing had already started. Administrative problems related to the import of the devices were encountered in Bangladesh, leading to delays in delivery of the devices. Therefore, initially, VIA for HPV-positive women in these countries was delayed until the AI-DSS was available.

#### Training

The initial training on how to use the AI-DSS was conducted online, as COVID-19 restrictions prevented the MAHE team from traveling to Bangladesh and Uganda. During the quarterly online project meetings, experiences with using the AI-DSS were shared, and concerns regarding the quality of the pictures were raised. During the project, the criteria for good-quality pictures for the AI-DSS were more clearly defined, and an update of the manual was produced. A troubleshooting section was added to the manual. More instructional videos were made available for health workers to address issues such as zooming and focusing. Additionally, instructions on saving patient data files were further specified. Additional instructional videos addressing specific UI issues were disseminated.

#### Implementation

The number of times VIA was performed and the AI-DSS was used varied from 136 times in Bangladesh to 1047 times in Uganda, while both countries tested around 6000 women for HPV. The prevalence of HPV infection (2/77, 2.6% in Bangladesh; 36/206, 17.5% in Uganda) had a large impact on how often the AI-DSS device was used.

The quality of a random sample of the pictures taken by health care workers was assessed by a panel of gynecologists, experts in VIA, and the MAHE team that designed the AI-DSS. In total, 38 of the 285 (13.3%) pictures that were scrutinized were rejected by the expert panels and/or the MAHE technical team (2/77, 2.6% in Bangladesh; 36/206, 17.5% in Uganda). In total, 20 pictures were rejected because of low quality: the picture was not sharp, had too much mucus, was not well colored with acetic acid, or was too dark. In total, 18 pictures were rejected because of unsatisfactory visibility of the cervix, especially the SCJ, due to insufficient zooming, poor positioning of the camera or speculum, or labia partly obscuring the vision of the SCJ. However, as the AI-DSS algorithm was calibrated to provide either a red (further investigation) or green (no anomalies) assessment, even low-quality pictures received an evaluation. Of the 38 rejected pictures, 52.6% (n=20) were red and 47.4% (n=18) were green.

The MAHE team remotely updated the software of the AI-DSS device when it was connected to a computer to enhance data exchange with the electronic medical record used in the project.

#### Monitoring

The implementation process was monitored through quarterly PRESCRIP-TEC consortium meetings, which were held online initially and transitioned to in-person meetings between months 24 and 36 of implementation. During the project, the research coordinator organized meetings of the panels of gynecologists, experts in VIA, to assess the pictures produced in the field. This continuous evaluation revealed issues with the quality of the pictures and led to further improvements in training and instructions. It also exposed the issue of performing VIA and operating the AI-DSS at the same time, as well as data communication issues.

#### Costs

In a separate study, the costs of VIA, including the use of AI-DSS in Uganda, were estimated at US $20.27, and in Bangladesh, US $5.57. The difference in costs is mainly explained by differences in personnel costs. In Uganda, higher costs for personnel were attributed to the presence of extra staff to operate the device [20].

### Barriers and Facilitators

Table 1 summarizes the barriers and facilitators in the 5 CFIR domains identified in the study.

**Table 1.** Facilitators and barriers for use of AI decision support systems for cervical cancer screening in Uganda and Bangladesh, as identified in this study, organized according to the Consolidated Framework for Implementation Research domains.

CFIR^a^ domain	Barriers	Facilitators
Innovation	Weight of the device generally requires a second person to operate. Process of transferring pictures to a computer is restricted. Zoom function is difficult to handle while holding the speculum.	Hardware is usable under resource-constrained conditions. The design of the UI^b^ is robust and intuitive. Manual with troubleshooting section and instructional videos is helpful.
Outer setting	Country regulations concerning AI-DSS^c^ are not in place. Funding for routine use of the device is not secured.	Health facilities are well equipped for gynecological examination. Electricity and internet for data storage are available.
Inner setting	Lack of regular supplies for cervical cancer screening.	AI-DSS operation can be easily integrated into routine VIA^d^.
Individuals	There are sometimes privacy concerns regarding pictures of women. Capabilities of health care workers may be limited.	Patients generally are satisfied with services. Training and preparation of staff are effective. Continuous supervision improves performance of VIA.
Process	AI-DSS does not recognize poor-quality pictures. AI-DSS does identify conditions for further examination, not precancerous lesions specifically.	Adjustments in the manual and training methods assist in improving performance. Remote updating of software enables improvement of performance. The operational costs are affordable.

a CFIR: Consolidated Framework for Implementation Research.

b UI: user interface.

c AI-DSS: AI decision support system.

d VIA: visual inspection with acetic acid.

## Discussion

### Principal Findings

This study provides insight into the implementation process of an AI-DSS for cervical cancer screening using VIA in 2 countries. In total, the AI-DSS was used 1183 times, and 285 pictures were analyzed, of which 38 were rejected due to quality issues. The device was easily accepted by health care workers and patients. Using the CFIR framework, with its 5 domains, barriers and facilitators were identified that are relevant to a smooth large-scale introduction of the device in cervical cancer screening (Table 1).

Under field conditions, the sensitivity and specificity of health care workers’ observations were slightly lower than those under laboratory conditions (assessing high-quality pictures on a computer screen). Time pressure and work stress during VIA may have contributed to lower accuracy. The AI-DSS was calibrated to find anomalies for further examination, and therefore, the negative predictive value was high (91.3%) but the positive predictive value was low (19.7%).

The rejection rate of pictures taken in Uganda (36/206, 17.5%) was much higher than in Bangladesh (2/77, 2.6%). This can be attributed to the less optimal preparation of health care workers in Uganda, as well as inadequate supervision in Uganda. After additional training, the rejection rate was reduced.

### Comparison to Prior Work

In recent years, several studies have been published about AI-DSS algorithms and their performance, including systematic reviews [24-28]. Research into the contextual factors, such as hardware used, UI, and human resources applying AI-DSS, however, is rare.

The use of the CFIR framework in this study helped identify relevant constructs across different domains (Multimedia Appendix 1) and provided an overview of barriers and facilitators (Table 1). The CFIR framework has been used in a few other studies for the analysis of AI-DSS in cervical cancer screening. One study in South Africa used the CFIR framework to compare 2 devices in a preclinical study at a research site [29]. Another study in Peru used the CFIR framework to evaluate the introduction of a device for remote and asynchronous expert colposcopist feedback (not AI-DSS) [30]. A third study in Uganda applied the CFIR framework in qualitative research to examine perceptions of stakeholders regarding the use of telemedicine and AI-DSS in cervical cancer screening [31]. According to these publications, the CFIR framework served as a solid and valuable framework for evaluating implementation research into AI-DSS in cervical cancer screening. This study confirms that the CFIR is a valuable tool for drawing lessons learned.

### Future Directions

This study is relevant because AI-DSS is likely to be used frequently in future health care [32], and it provides insights into the user experiences of the UI of medical devices [33].

The findings of this research provide important lessons for introducing AI-DSS in cervical cancer screening in LMICs. Although the hardware (modified mobile phone) was equipped with a good camera, a clear screen, and sufficient lighting necessary for an AI application [34], a smaller and lighter type of device may have been preferable, just as fixing the device on a holder attached to the speculum would be helpful to secure its position, distance, and location vis-à-vis the cervix. Alternatively, a device on a tripod could be considered, which can be positioned after the cervix is visualized. Integrating the device into an ablation device could be another option that would allow for operation by a single health care worker. The offline mode of the device proved very useful, as the evaluations in Uganda and Peru highlighted issues with connectivity that prevented access to the algorithm. Additionally, using the mobile phone’s battery for lighting and picture capturing proved very helpful in field conditions without regular electricity.

Elhaddad and Hamam [32] state that UI design must be user-centered, fit into the user’s workflow, and be trusted, addressing attitudinal barriers among health care workers. The design of the SAKHI Manipal UI was indeed user-centered, guiding the health care worker through the workflow of cervical cancer screening. In general, the device’s use was intuitive. However, the zoom function could be made easier to operate when one person takes the picture while performing VIA.

The capacities of health care workers are crucial in operating the AI-DSS. Even if the task-shifting mode of the AI-DSS is used, the health care worker must be capable of performing VIA, including coloring, cleaning the cervix, and positioning the speculum correctly before taking the picture. Clear visibility of the SCJ is a precondition for using the AI-DSS. For future use in field conditions, it would be beneficial if the AI-DSS provided a mode that could indicate the quality of the picture before assessment, for example, by producing an orange traffic light that forces the provider to retake the picture.

The device produced a folder with a random number of the patient and pictures for further evaluation and research, which was very helpful in the research project. Discrepancies in assessment by health care workers and by AI-DSS led to discussions during supervision visits. Continuously comparing the AI-DSS assessment with that of the health care workers builds capacity and trust in the device.

The pictures were also used for communication with a supervisor working remotely. In this case, the limitations of the device with disabled Wi-Fi and SIM were a disadvantage, leading to delayed communication. The future introduction of passwords or facial recognition could facilitate wireless communication between devices, such as a phone and another phone or tablet.

Reassuring the patient about the confidentiality of the pictures and showing the pictures could be part of the standard procedure when using cameras for AI-DSS.

Future integration into regular screening procedures appears to be feasible, as it aligns with existing practice and does not necessitate a separate intervention. However, trust in the AI-DSS remains crucial. Both health workers and patients must be confident that technology can provide a reliable diagnosis. The algorithm used in our project requires further development to increase the accuracy of the AI-DSS and differentiate between precancerous lesions and other anomalies. Governments must develop clear policies and regulations concerning the use of AI in diagnostics, including procedures for task shifting (initial assessment by AI-DSS) and addressing disagreements between AI and health care workers. The costs of the device (now US $600) can be reduced in the future as mass production starts. Another solution is to combine medical apps, such as those for obstetrics and ultrasound, on a single device. Given the privacy issues, it is not recommended to install the software on a regular phone used by health care workers.

Overall, the study employed a process of learning by doing and identified issues and shortcomings that were subsequently addressed. This is an essential attitude in implementation research, which focuses on improving practice [35]. Our monitoring process was therefore successful.

### Limitations of This Research

First, in evaluating the introduction of the AI-DSS, we employed various data collection methods, as described in the methodology section. Therefore, this study presents a secondary analysis of data collected for various purposes, including patient satisfaction, user satisfaction, and project monitoring. The CFIR constructs could have been formulated more clearly upfront.

Second, we did not interview all health care workers who used the device and therefore may have missed viewpoints. Minutes of quarterly evaluation meetings and reports of supervision visits were used for monitoring. No transcripts of meetings were made, and relevant points may therefore have been overlooked. Better reporting formats could have been applied.

Third, the introduction took place in a research environment, and task shifting was not applied because of ethical considerations. Research into the real-life use of the device could reveal how disagreements in assessment between a health care worker and AI-DSS can be resolved. We were unable to measure the long-term impact of the AI-DSS on the prevention of cervical cancer [35]. Only after registration of the device as a medical device can the impact of task shifting be measured.

Fourth, due to COVID-19–related restrictions, initial capacity building was conducted online, which may have impacted the quality of training for health care workers. Later, face-to-face training was applied.

### Recommendations

Introduction of AI-DSS in cervical cancer screening in low-resource settings is part of the future, certainly for cervical cancer screening using VIA [36]. Further research is needed into the facilitators and barriers when the AI-DSS operates in a routine cervical cancer screening program. Further research into the potential for task shifting once the AI-DSS device is licensed as a medical device is also recommended.

### Conclusion

This study demonstrates that the introduction of an AI-DSS necessitates a broad spectrum of interventions and preconditions. We identified a suitable hardware device and developed a user-centered, intuitive UI that follows the workflow of health care workers. We recognize that software adaptations are required to increase the accuracy of the algorithm.

We showed that capacity building is crucial for the successful implementation of AI-DSS. Health care workers must be capable of performing a high-quality VIA and must be able to take excellent pictures that can be fed to the AI-DSS algorithm. We showed that the workload will not increase when AI-DSS is applied routinely.

We conclude that an AI-DSS device has the potential to assist health workers in better decision-making but requires careful introduction. Further research under field conditions is necessary.

## Supplementary material

10.2196/81493 Multimedia Appendix 1 Sources of information per domain.

10.2196/81493 Multimedia Appendix 2 Confusion matrix tables.

10.2196/81493 Multimedia Appendix 3 Details of quantitative information used from surveys.

## Abbreviations

CFIR: Consolidated Framework for Implementation Research
DSS: decision support system
HPV: human papillomavirus
hrHPV: high-risk HPV
LMIC: low- and middle-income country
MAHE: Manipal Academy of Higher Education
NGO: nongovernmental organization
PRESCRIP-TEC: Prevention and Screening Innovation Project Towards Elimination of Cervical Cancer
SCJ: squamous-columnar junction
UI: user interface
VIA: visual inspection with acetic acid
WHO: World Health Organization
