# Supplementary material for: Applying an AI Decision Support Device During Cervical Cancer Screening in Bangladesh and Uganda: Implementation Study
Source: JMIR Form Res. 2026 Sep 3;10:e81493. doi: 10.2196/81493 (PMC13541229; doi:10.2196/81493)
Supplement: Supplementary file 1 [file formative-v10-e81493-s001.docx]

**Applying a Device for Artificial Intelligence Decision Support in practice during screening for cervical cancer in Bangladesh and Uganda; a CFIR analysis**

**Supplementary file 1** data sources that identified for each of the domains.

| **Domains** | **Constructs in domains** | **Data source**s^[1]^ |
| --- | --- | --- |
| **Innovation** | Evidence base | Scientific publications (12) |
|  | Relative advantage | Scientific publications (3, 6, 13) |
|  | Design of Hardware | Scientific publications (12) |
|  | Design of user interface | SAKHI manual (22) |
|  | Costs | PRESCRIP-TEC finances |
|  | Triability | Interviews with stakeholders |
| **Outer setting** | Regulations | International regulations for medical devices (23) |
|  | Local conditions | SARA survey in facilities |
|  | Funding | Cost effectiveness study PRESCRIP-TEC (16, |
| **Inner setting** | Structural characteristics | Routine data (SF2) |
|  | Communications | Interviews |
|  | Mission alignment | SARA survey PRESCRIP-TEC |
| **Individuals** | Recipients (patients) | AWACAN survey, HSTF survey,  Pre- post-survey patients (AF2) |
|  | Implementing team | Interviews with health workers, minutes meetings |
|  | Supervisors | Interviews with health workers, minutes meetings |
|  | Technical support team | Interviews with health workers, minutes meetings |
| **Process** | Preparation | Scientific publications (20, 21) |
|  | Training | Evaluation survey PRESCRIP-TEC |
|  | Implementation | AI-DSS data  Gynecological panel assessments |
|  | Monitoring | AI-DSS data  EMR data |
|  | Operational costs | Cost effectiveness study PRESCRIP-TEC |

Table additional file 1 CFIR domains and constructs in each of the domains and sources of information per domain of specific research activities during this study into the use of an AI-DSS device within the PRESCRIP-TEC research project into cervical cancer screening in Uganda and Bangladesh.

1. Numbers in brackets refer to publication in references. SF2 stands for Supplementary File 2.
