# Supplementary material for: Applying an AI Decision Support Device During Cervical Cancer Screening in Bangladesh and Uganda: Implementation Study
Source: JMIR Form Res. 2026 Sep 3;10:e81493. doi: 10.2196/81493 (PMC13541229; doi:10.2196/81493)
Supplement: Supplementary file 2 [file formative-v10-e81493-s002.docx]

**Applying a Device for Artificial Intelligence Decision Support in practice during screening for cervical cancer in Bangladesh and Uganda; a CFIR analysis**

**Supplementary file 3 Confusion matrixes**

Sensitivity and specificity and Cohen’s Kappa of both AI-DSS and health workers assessment

| **Sensitivity and Specificity** | | | | |
| --- | --- | --- | --- | --- |
| **AI DSS in Uganda and Banladsh** | | | | |
|  | Gyn Panel  VIA pos | Gyn Panel  VIA neg | Totals |  |
| AI-DSS VIA pos | 17 | 69 | 86 |  |
| AI-DSS VIA neg | 14 | 147 | 161 |  |
|  |  |  |  |  |
| Totals | 31 | 216 | 247 |  |
|  | | | |  |
| Accuracy | 66,40% |  |  |  |
| Sensitivity AI-DSS | 54,84% |  |  |  |
| Specificy AI-DSS | 68,06% |  |  |  |
| Pos Pred Value AI-DSS | 19,77% |  |  |  |
| Neg Pred Value AI-DSS | 91,30% |  |  |  |
| Cohen‘s Kappa | 0,13 |  |  |  |

| **Heatlh workers in Uganda and Bangladesh** | | | |
| --- | --- | --- | --- |
|  | Gyn Panel  VIA pos | Gyn Panel  VIA neg | Totals |
| HW VIA pos | 20 | 45 | 65 |
| HW VIA neg | 11 | 171 | 182 |
|  |  |  |  |
| Totals | 31 | 216 | 247 |
|  | | | |
| Accuracy | 77,33% |  |  |
| Sensitivity HW VIA | 64,52% |  |  |
| Specificy HW VIA | 79,17% |  |  |
| Pos Pred Value HW | 30,77% |  |  |
| Neg Pred Value HW | 93,96% |  |  |
| Cohen’s Kappa | 0,30 |  |  |

| Agreement between Gyn Panel, AI and HW | |  | Number | Percentage |
| --- | --- | --- | --- | --- |
| All agree (both pos and neg VIA) | |  | 130 | 52,63% |
| AI and GP agree, not health worker | |  | 34 | 13,77% |
| HW and GP agree not AI | |  | 61 | 24,70% |
| HW and AI agree not GT | |  | 22 | 8,91% |
|  |  |  | 247 | 100,00% |
