# Supplementary material for: Applying an AI Decision Support Device During Cervical Cancer Screening in Bangladesh and Uganda: Implementation Study
Source: JMIR Form Res. 2026 Sep 3;10:e81493. doi: 10.2196/81493 (PMC13541229; doi:10.2196/81493)
Supplement: Supplementary file 3 [file formative-v10-e81493-s003.docx]

**Applying a Device for Artificial Intelligence Decision Support in practice during screening for cervical cancer in Bangladesh and Uganda; a CFIR analysis**

Supplementary file 3

**Extract from Routine data HPV test and VIA**

### Bangladesh:

- Number of women actively invited for self-collected swab: **7.900**
- Number of women who have done a self-collected swab: **7.831**
- Number of lab tests done: **8.323** – including quality control, retesting, invalids
- Number of HPV positive women: **164** - 2,1% of women who did a self-collected swab
- Number of VIA: **136** - 82,9% of HPV positive women
- Number of VIA positive: **55** - 40,4% of VIA
- Number of women treated: **51** - 92,7% of VIA positive
- Number of referrals: **10** – 7,4% of VIA

### Uganda:

- Number of women actively invited for self-swab: **7.126**
- Number of women who have done a self-swab: **7.124**
- Number of lab tests done: **7.399** -– including quality control, retesting, invalids.
- Number of HPV positive women: **1.606** - 22,5% of women who did a self-swab
- Number of VIA: **1.047** - 65,2% of HPV positive women
- Number of VIA positive: **159** - 15,2% of VIA
- Number of women treated: **1.043** - 99,6% of VIA as national policy is to treat all HPV positive
- Number of referrals: **23** - 2,2% of VIA - made by CHWs due to suspected cervical cancer and absence of SCJ.

**AWACAN survey**

Methodology see: https://awacan.online

**Bangladesh**

| Variables | Women 30-60 years  (n=600), n (%) | Household decisionmakers  (n=1200), n (%) | |
| --- | --- | --- | --- |
|  |  | Husband (n=600), n (%) | Mother-in-law (n=600), n (%) |
| Mean age (years) ± SD | 37±5.33 | 43±6.62 | 65±9.08 |
| Religion | | | |
| Islam | 554 (92.3) | 557 (92.8) | 555 (92.5) |
| Hinduism | 46 (7.7) | 43 (7.2) | 45 (7.5) |
| Education | | | |
| Below primary | 339 (56.5) | 386 (64.3) | 584 (97.3) |
| Primary | 225 (37.5) | 152 (25.3) | 16 (2.7) |
| Secondary | 19 (3.2) | 43 (7.2) | 0 |
| Higher secondary and above | 17 (2.8) | 19 (3.2) | 0 |
| Occupation | | | |
| Housewife | 568 (94.7) | 0 (0) | 592 (98.7) |
| Day labor | 11 (1.8) | 111 (18.5) | 5 (0.8) |
| Farmer/Fisherman/Boatman | 2 (0.3) | 375 (62.5) | 3 (0.5) |
| Business | 11 (1.8) | 80 (13.3) | 0 |
| Others | 8 (1.4) | 34 (5.7) | 0 |
| Monthly family expenditure (BDT & USD) | | | |
| 0-5000 (0-52 USD) |  | 11 (1.8) | 51 (8.5) |
| 5001-10000 (52-105 USD) |  | 348 (58.0) | 337 (56.2) |
| 10001-20000 (105-210 USD) |  | 212 (35.3) | 189 (31.5) |
| >20000 (>210 USD) |  | 29 (4.8) | 23 (3.8) |
| Information on knowledge of cervical cancer and screening history | | | |
| Ever heard of cervical cancer | 486 (81.0) | 406 (67.7) | 445 (74.2) |
| Ever tested for cervical cancer | 37 (6.2) |  |  |
| Last tested for cervical cancer |  |  |  |
| >5 years ago | 24 (64.9) |  |  |
| 3-5 years ago | 2 (5.4) |  |  |
| 1-2 years ago | 7 (18.9) |  |  |
| <1 year ago | 4 (10.8) |  |  |
| Decisions regarding women's health | | | |
| Myself | 20 (3.3) |  |  |
| Partner | 244 (40.7) |  |  |
| Myself and partner jointly | 265 (44.2) |  |  |
| Someone else in household | 21 (3.5) |  |  |
| Myself and someone else jointly | 61 (10.2) |  |  |

Uganda

| **Variable** | Category | Female N = 724 | Male N = 692 |
| --- | --- | --- | --- |
| Age (years) | Mean (SD) | 37.39 (5.69) | 37.88 (7.13) |
|  |  | **n (%)** | **n (%)** |
| Education | No education | 69 [16.2] | 71 [16.1] |
|  | Primary School | 325 [76.5] | 335 [75.7] |
|  | Secondary school | 31 [7.3] | 33 [7.5] |
|  | Tertiary | 0 | 3 [0.7] |
| Marital status | Married | 312 [63.3] | 272 [ 62.0] |
|  | Single | 48 [9.8] | 56 [ 12.8] |
|  | Widowed | 17 [3.4] | 13 [ 3.0] |
|  | Cohabiting | 107 [21.7] | 90 [ 20.5] |
|  | Divorced | 9 [1.8] | 8 [ 1.7] |
| Ever heard of cervical cancer? | Yes | 510 [ 71.0] | 459 [67.2] |
|  | No | 208 [ 29.0] | 224 [ 32.8] |
| Has a health worker ever tested you for cervical cancer? | Yes | 61 [ 8.8] | NA |
|  | No | 633 [91.0] | NA |
|  | Refused | 1 [ 0.1] | NA |
|  | Don’t know | 1 [0.1] | NA |
| Who makes decisions concerning your health? | Myself | 229 [31.7] | NA |
|  | My partner | 254 [ 35.2] | NA |
|  | Myself and partner jointly | 226 [31.8] | NA |
|  | Someone else in the household | 11 [ 1.5] | NA |
|  | Myself and someone else jointly | 2 [ 0.3] | NA |

**Health Systems Trust Framework**

Methodology: See

Bangladesh:

Trust in health workers

“ I believe my healthcare providers are technically competent”

|  | Very negative | Negative | Neutral | Positive | Very positive | Total |
| --- | --- | --- | --- | --- | --- | --- |
| Women | 0 | 3 | 15 | 409 | 596 | 1023 |
| Men | 1 | 0 | 6 | 111 | 157 | 275 |
| Family members (decision-makers) | 0 | 2 | 6 | 109 | 121 | 238 |
| Totals | 1 | 5 | 27 | 629 | 874 | 1536 |
| Percentage | 0.1 | 0.3 | 1.8 | 41.0 | 56.9 | 100 |

Trust in therapy

“I believe that my healthcare provider will give me the right treatment”

|  | Very negative | Negative | Neutral | Positive | Very positive | Total |
| --- | --- | --- | --- | --- | --- | --- |
| Women | 0 | 1 | 12 | 468 | 542 | 1023 |
| Men | 1 | 0 | 10 | 123 | 141 | 275 |
| Family members (decision-makers) | 1 | 0 | 9 | 129 | 99 | 238 |
| Totals | 2 | 1 | 31 | 720 | 782 | 1536 |
| Percentage | 0.1 | 0.1 | 2.0 | 46.9 | 50.9 | 100 |

**Uganda**

Trust in health workers

“ I believe my healthcare providers are technically competent”

|  | Very negative | Negative | Neutral | Positive | Very positive | Total |
| --- | --- | --- | --- | --- | --- | --- |
| Women | 0 | 1 | 7 | 215 | 13 | 236 |
| Men |  | 0 | 7 | 199 | 19 | 225 |
| Totals | 0 | 1 | 14 | 414 | 31 | 461 |
| Percentage | 0% | 0.2% | 3.0% | 89,8% | 6.7% | 100% |

Trust in therapy

“I believe that my healthcare provider will give me the right treatment”

|  | Very negative | Negative | Neutral | Positive | Very positive | Total |
| --- | --- | --- | --- | --- | --- | --- |
| Women | 0 | 0 | 9 | 211 | 15 | 235 |
| Men | 0 | 2 | 6 | 197 | 20 | 225 |
| Totals | 0 | 2 | 15 | 408 | 35 | 460 |
| Percentage | 0% | 0.4% | 3 | 88.6% | 7.6% | 100% |

Trust in AI among women tested

|  | Very negative | Negative | Neutral | Positive | Very positive | Don’t know | Total |
| --- | --- | --- | --- | --- | --- | --- | --- |
| Numbers | 2 | 119 | 5 | 110 | 17 | 6 | 259 |
| Percentage | 0.8 | 45.9 | 1.9 | 42.5 | 6.6 | 2.3 | 100 |
